# Expression of anaesthetic and analgesic drug target genes in excised breast tumour tissue: Association with clinical disease recurrence or metastasis

**DOI:** 10.1371/journal.pone.0177105

**Published:** 2017-05-30

**Authors:** C. Connolly, S. F. Madden, D. J. Buggy, H. C. Gallagher

**Affiliations:** 1 Dept. of Anaesthesia, Mater Misericordiae University Hospital, Dublin, Ireland; 2 School of Medicine, University College Dublin, Dublin, Ireland; 3 RCSI Population Health Sciences, Dept. of Psychology, Royal College of Surgeons, Dublin, Ireland; 4 Conway Institute for Biomolecular and Biomedical Research, University College Dublin, Dublin, Ireland; 5 Outcomes Research Consortium, Cleveland Clinic, Cleveland, Ohio, United States of America; National Cancer Center, JAPAN

## Abstract

**Background:**

Retrospective analyses suggest anaesthetic-analgesics technique during cancer surgery may affect recurrence/metastasis. This could involve direct effects of anaesthetic-analgesic drugs on cancer cells. While μ-opioid receptor over-expression in lung tumours is associated with greater metastasis, other anaesthetic-analgesic receptor targets in cancer recurrence/metastasis remain unexplored. Therefore, we evaluated the association between genetic expression of anaesthetic-analgesic receptor targets and recurrence/metastasis, using a repository of breast cancer gene expression and matching clinical data.

**Methods:**

A list of 23 genes encoding for the most prominent anaesthetic-analgesic receptor targets was compiled. This was processed through *BreastMark*- an algorithm integrating gene expression data from ~17,000 samples and clinical data from >4,500 breast cancer samples. Gene expression data was dichotomized using disease-free survival (survival without recurrence) and distant disease-free survival (survival without metastasis) as end points. Hazard ratios were calculated by Cox-regression analysis. Enrichment for prognostic markers was determined by randomly choosing 23-member gene lists from all available genes, calculating how often >5 significant markers were observed and adjusting p-values for multiple testing. This was repeated 10,000 times and an empirical p-value calculated.

**Results:**

Of 23 selected genes, 9 were significantly associated with altered rates of metastasis and 4 with recurrence on univariate analysis. Adjusting for multiple testing, 5 of these 9 genes remained significantly associated with metastasis, non with recurrence. This ratio of genes (5/23) was not significantly enriched for markers of metastasis (p = 0.07).

**Conclusion:**

Several anaesthetic-analgesic receptor genes were associated with metastatic spread in breast cancer. Overall there was no significant enrichment in prognostic markers of metastasis, although a trend was observed.

## Introduction

Despite the continuing introduction of novel treatment strategies, breast cancer remains a leading cause of death in women. It is not usually the primary breast tumour, but rather metastasis at distant sites that cause mortality [1]. Therefore, any potential strategy to decrease the progression of primary breast cancer to metastatic disease warrants investigation.

A number of perioperative factors have been hypothesized to affect metastasis after cancer surgery including anaesthetic-analgesic technique [2, 3]. Some retrospective clinical analyses have found an association between perioperative use of volatile anaesthetics and opiate analgesics and increased risk of subsequent metastases. This may reflect transitory host immune suppression, but recent experimental studies also indicate that anaesthetics and analgesic drugs themselves directly influence cancer cell biology, in ways which can promote or resist metastasis [3, 4].

Therefore, it is plausible that perioperative drugs, given during cancer surgery, modulate cancer cell biology by directly binding to receptors in tumours. A corollary to this hypothesis is that the level of receptor expression in a given tumour may influence how responsive those tumour cells are to individual drugs acing on receptors. Available experimental evidence suggests that use of some anaesthetic-analgesic agents is associated with reduced cancer metastasis (e.g. local anaesthetics, propofol) [5, 6], while others may be associated with increased risk of metastasis (e.g. volatile agents, opioids) [7, 8]. Indeed, an association between over-expression of the μ opioid receptor (MOR) protein and increased incidence of lung cancer metastasis has been identified [9]. However, there is much conflicting evidence and genetic expression of anaesthetic and analgesic target genes within tumour tissue has never been investigated with respect to longitudinal clinical outcomes.

This retrospective analysis tests the hypothesis that gene expression levels of certain anaesthetic and analgesic receptor targets in breast cancer tissue are associated with either increased or decreased incidence of metastasis and local recurrence. It employs an online, publically accessible database of gene expression data from breast cancer patients (*BreastMark*) [10].

## Methods

We interrogated *BreastMark—*an algorithm that is freely available online that integrates published gene expression data (corresponding to ~17,000 genes and 341 miRNAs) and detailed clinical data relating to outcome in breast cancer. It contains transcriptomic and clinical information derived from 26 microarray datasets, across 12 different array platforms, incorporating 4,738 patient samples. This database has been extensively validated by reference to the MammaPrint [11] and oncotype Dx [12] gene signatures and only includes high quality microarray studies. It employs an algorithm that combines gene expression data from multiple microarray experiments and detailed clinical data to correlate outcome with gene expression levels. As multiple platforms are combined by BreastMark, and not all platforms contain probes for all genes, the number of samples in a comparison will vary depending on how many platforms have probes for the gene of interest. For more details on this algorithm visit http://glados.ucd.ie/BreastMark/index.html [10].

### Selection of target genes

We compiled a list of anaesthetic and anaesthetic target genes, which was used to interrogate the *BreastMark* database. These receptors comprised the main targets for anaesthetic and opioid analgesic drugs that are commonly used in the peri-operative period. While opioids bind to single polypeptide G-protein-coupled receptors, most anaesthetic receptors are ion channels and they therefore comprise multiple peptide subunits and are coded for by more than one gene. Therefore, we concentrated on those genes that encode receptor peptide subunits for which confirmed anaesthetic binding sites have been identified. This was based on literature searching of X-ray crystallography, mutagenesis and receptor binding affinity studies. A list of 23 genes was created, reflecting the verified major anaesthetic and opioid analgesic receptor protein binding sites (Table 1).

**Table 1:** List of 23 genes that encode for the receptor proteins that interact with the major anaesthetic and opioid analgesic receptors for which gene expression data was determined in the *BreastMark* database.

Anaesthetic/Analgesic Receptor-Protein Target	Official HGNC Symbol	Entrez Gene ID	Site and mode of Anaesthetic/AnalgesicAction
Delta Opioid receptor (DOR)	OPRD1	4985	Positively modulated by opioids.
Mu Opioid receptor (MOR)	OPRM1	4988	Positively modulated by opioids.
Kappa Opioid receptor	OPRK1	4986	Positively modulated by opioids.
NMDA receptor subunit ‘GRIN1’	GRIN1	2902	Inotropic glutamate receptor activated by glycine.
NMDA receptor subunit ‘GRIN2A’	GRIN2A	2903	Inhibited by volatile anaesthetics & xenon.
NMDA receptor subunit ‘GRIN2B’	GRIN2B	2904	Inhibited by volatile anaesthetics & xenon.
NMDA receptor subunit ‘GRIN2C’	GRIN2C	2905	Inhibited by volatile anaesthetics & xenon.
NMDA receptor subunit ‘GRIN3A’	GRIN3A	116443	Inhibited by volatile anaesthetics.
NMDA receptor subunit ‘GRINA’	GRINA	2907	Modulates function via glycine, rather than NMDA compound. Therefore more affected by propofol and IV anaesthesia.
Noradrenaline channel transporter	SLC6A2	6530	Sodium-dependent noradrenaline reuptake, inhibited by propofol.
5HT channel transporter	SLC6A4	6532	Sodium-dependent serotonin reuptake, inhibited by volatile anaesthetics.
Glycine receptor α1subunit	GLRA1	2741	Positively modulated by propofol and volatile anaesthetics.
Glycine receptor β subunit	GLRB	2743	Positively modulated by propofol.
GABA_A_ receptor subunit α1	GABRA1	2554	Positively modulated by volatile anaesthetics.
GABA_A_ receptor subunit α2	GABRA2	2555	Positively modulated by volatile anaesthetics.
GABA_A_ receptor subunit α3	GABRA3	2556	Positively modulated by volatile anaesthetics.
GABA_A_ receptor subunit α5	GABRA5	2558	Positively modulated by volatile anaesthetics.
GABA_A_ receptor subunit β1	GABRB1	2560	Positively modulated by propofol and volatile anaesthetics.
GABA_A_ receptor subunit β2	GABRB2	2561	Positively modulated by propofol and volatile anaesthetics.
GABA_A_ receptor subunit β3	GABRB3	2562	Positively modulated by propofol and volatile anaesthetics.
GABA_A_ receptor subunit γ1	GABRG1	2565	Positively modulated by propofol & barbiturates.
GABA_A_ receptor subunit γ2	GABRG2	2566	Positively modulated by propofol & barbiturates.
GABA_A_ receptor subunit γ3	GABRG3	2567	Positively modulated by volatile anaesthetics.

List of 23 genes that encode for the receptor proteins that interact with the major anaesthetic and opioid analgesic receptors for which gene expression data was determined in the *BreastMark* database.

### Analysis of target genes in BreastMark

The 23 anaesthetic and analgesic target genes were analysed with *BreastMark* by dichotomizing gene expression data around a median into ‘high’ and ‘low’ expression, with 50% of patient samples in each group. Kaplan Meier survival curves were generated comparing these two populations (high and low expressers) for each gene for both disease-free survival (DFS; i.e. local recurrence) and distant disease-free survival (DDFS; i.e. metastasis). Cox regression analysis was used to generate the associated hazard ratios (HR) and confidence intervals (CI). This approach has previously been employed to validate the *BreastMark* database in confirming prognostic signatures associated with the commercially available Mammaprint and Oncotype Dx diagnostic tests for breast cancer [10].

### Screening of gene list for the prognostic potential in breast cancer using *BreastMark*

Using *BreastMark* we performed a screen of the prognostic potential of all available genes to determine if our selected group of 23 genes was enriched for prognostic markers. Gene expression data was dichotomized around median expression, dividing our population into ‘high’ and ‘low’ expressers of target genes. DDFS (reflecting survival without metastasis) was used as the primary survival end-point. DFS (reflecting local recurrence) was also determined for those genes whose expression showed an association with metastasis. Kaplan-Meier estimates and the log-rank *p*-value indicated differences in survival. *P*-values were adjusted for multiple testing using the Benjamini-Hochberg method [13] with *p*-values less than 0.05 considered significant. Cox regression analysis was used to calculate HRs and CIs. Enrichment for prognostic markers was determined by randomly choosing gene lists of identical size to our own list (23) and calculating how often the same number or more significant prognostic markers were observed and at each stage adjusting the p-values using the same method as mentioned above (5 of our 23 genes were significant after adjusting for multiple testing). This was repeated 10,000 times and an empirical *p*-value was calculated.

## Results

### Association between target gene expression and DDFS (metastasis) or DFS (local recurrence)

**Fig 1:**
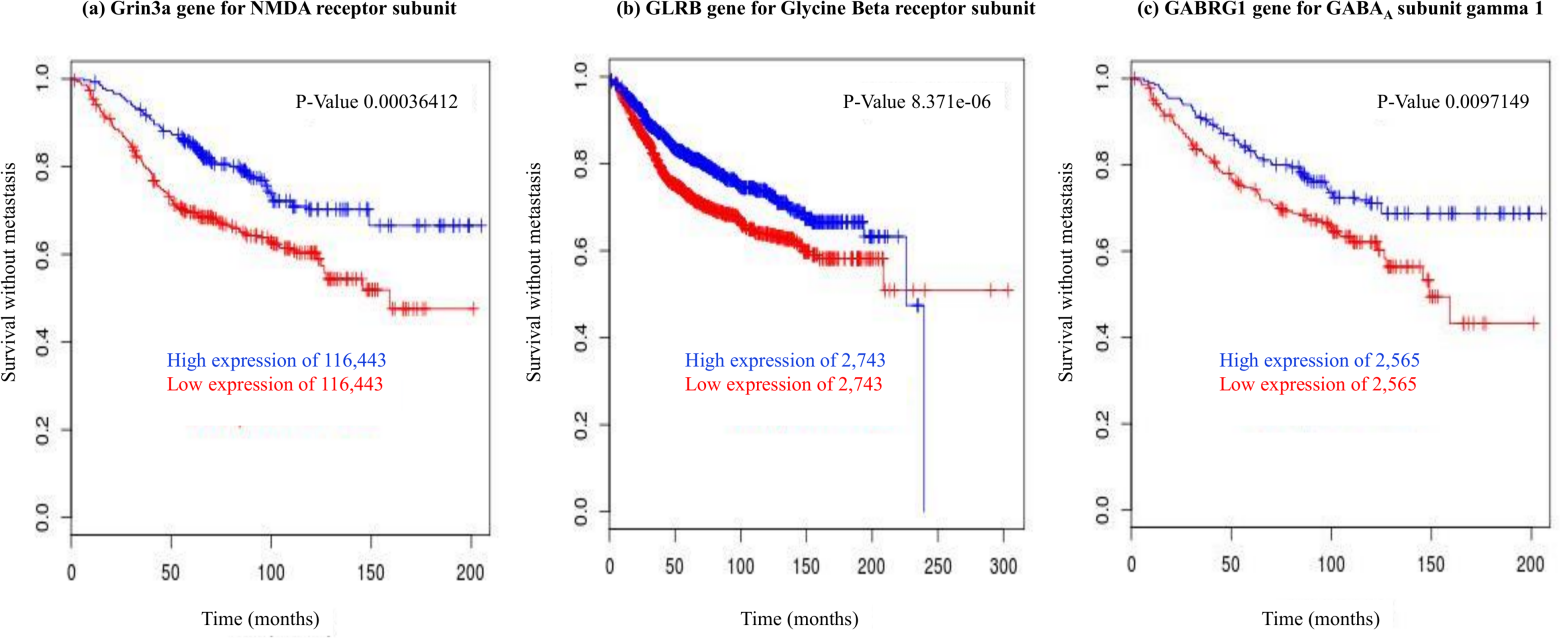
Kaplan-Meier plots for anaesthetic and analgesic targets where high gene expression is associated with reduced time to metastasis, presented in order of significance. Samples in the *Breastmark* database were dichotomized for gene expression around a 50% median value and differences between these two populations are indicated by hazard ratios. P-values shown are those obtained by univariate analysis of the Breastmark database for the indicated gene. Adjusted p-values were calculated on the basis of adjusting for multiple testing as described in Methods. A: GRINA; B: Noradrenaline transporter; C: Mu opioid receptor; D: Delta opioid receptor; E: GABA_A_ receptor γ3.

**Fig 2:**
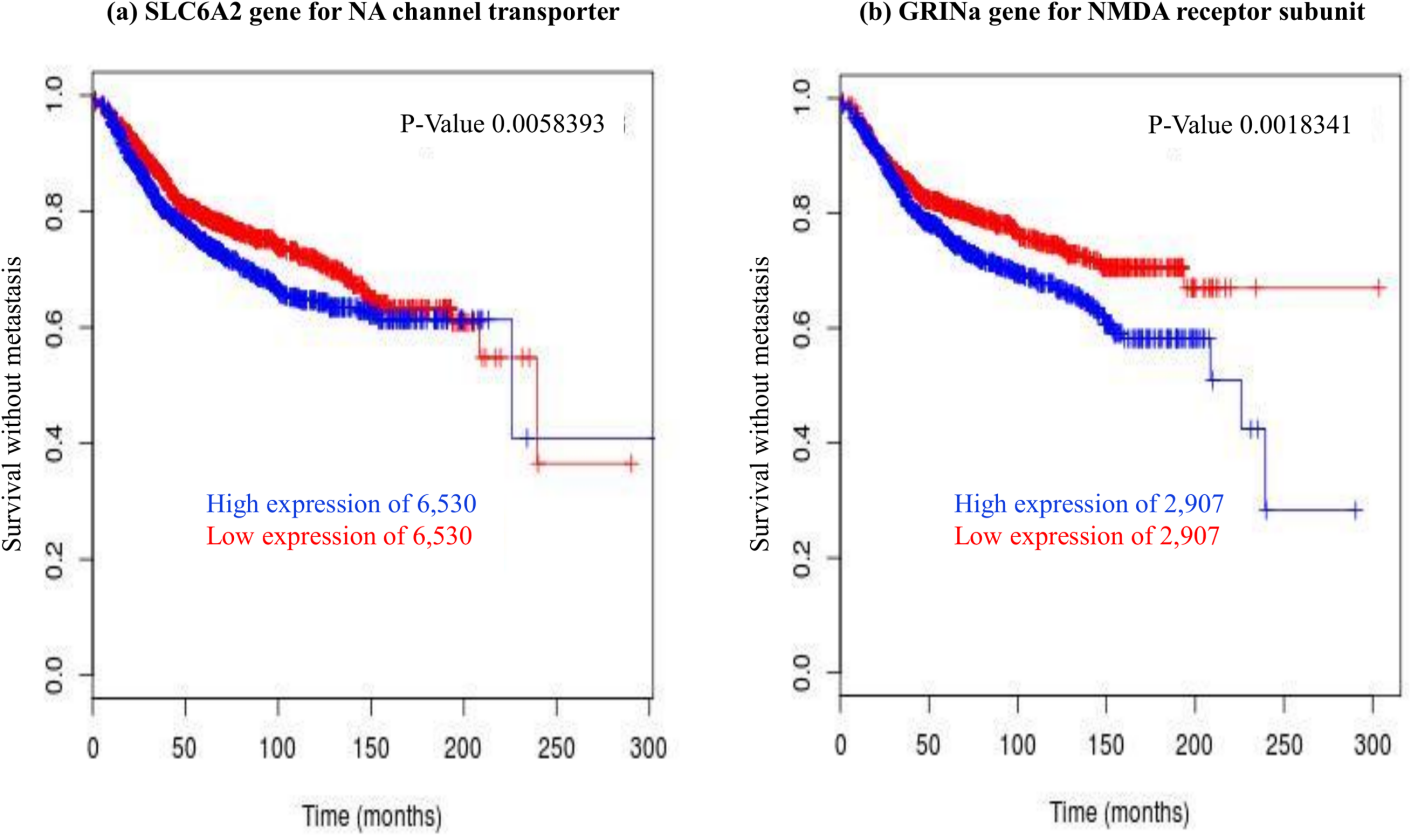
Kaplan-Meier plots for anesthetic and analgesic targets where low expression is associated with reduced time to metastasis, presented in order of significance. Samples in the *Breastmark* database were dichotomized for gene expression around a 50% median value and differences between these two populations are indicated by hazard ratios. P-values shown are those obtained by univariate analysis of the Breastmark database for the indicated gene. Adjusted p-values were calculated on the basis of adjusting for multiple testing, as described in Methods. A: Glycine beta receptor; B: GRIN3A; C: GABA_A_ receptor γ1; D: 5HT Transporter.

Of the chosen list of 23 genes, nine were associated with metastasis (*p*<0.05; Table 2; Figs 1 and 2) and four were associated with local recurrence (data not shown) when analysis of the *Breastmark* database was conducted. However, when we adjusted for multiple testing, only 5 genes remained statistically associated with metastasis on the basis of corrected *p*-values of <0.05 (Figs 1 and 2; Table 2) and none were associated with local recurrence (Table 3).

**Table 2:** Hazard ratios for anaesthetic and analgesic target genes based on the *BreastMark* database.

Anaesthetic/Analgesic Receptor Gene Target	Distant disease-free survival (metastasis)				
	Hazard Ratio (95% Confidence Intervals)	P-value	Adjusted P-Value	Sample Number	Events Number
Delta Opioid receptor (DOR)	1.21 (1.01–1.45)	0.04*	0.12	2223	593
Mu Opioid receptor (MOR)	1.20 (1.02–1.42)	0.03*	0.10	2223	593
Kappa Opioid receptor	1.16 (0.96–1.39)	0.12	0.25	2223	593
NMDA receptor subunit ‘GRIN1’	1.13 (0.96–1.33)	0.14	0.25	2223	593
NMDA receptor subunit ‘GRIN2A’	1.05 (0.84–1.27)	0.54	0.69	2223	593
NMDA receptor subunit ‘GRIN2B’	0.95 (0.79–1.15)	0.62	0.75	2223	593
NMDA receptor subunit ‘GRIN2C’	1.05 (0.89–1.25)	0.53	0.69	2223	593
NMDA receptor subunit ‘GRIN3A’	0.59 (0.44–0.79)	4.0 x 10^−4^*	4.6 x 10^−3^*	577	187
NMDA receptor subunit ‘GRINA’	1.35 (1.12–1.64)	1.8 x 10^−3^*	0.01*	1736	428
Noradrenaline channel transporter	1.25 (1.07–1.47)	6 x 10^−3^*	0.05*	2223	593
5HT channel transporter	0.83 (0.70–0.98)	0.03*	0.10	2223	593
Glycine receptor α1 subunit	1.09 (0.90–1.33)	0.37	0.61	2223	593
Glycine receptor β subunit	0.69 (0.59–0.81)	8.4 x 10^−6^*	1.9 x 10^−4^*	2223	593
GABA_A_ receptor subunit α1	1.10 (0.84–1.23)	0.89	0.92	2223	593
GABA_A_ receptor subunit α2	1.06 (0.89–1.28)	0.50	0.69	2223	593
GABA_A_ receptor subunit α3	1.01 (0.83–1.24)	0.91	0.92	2069	546
GABA_A_ receptor subunit α5	1.14 (0.96–1.36)	0.13	0.25	2223	593
GABA_A_ receptor subunit β1	0.92 (0.76–1.13)	0.43	0.66	2223	593
GABA_A_ receptor subunit β2	0.86 (0.72–1.03)	0.10	0.23	2223	593
GABA_A_ receptor subunit β3	1.04 (0.86–1.24)	0.71	0.78	2223	593
GABA_A_ receptor subunit γ1	0.64 (0.46–0.90)	0.01*	0.05*	423	140
GABA_A_ receptor subunit γ2	0.96 (0.79–1.16)	0.66	0.76	2223	593
GABA_A_ receptor subunit γ3	1.20 (1.00–1.44)	0.05*	0.13	2223	593

Samples were dichotomized for gene expression around a 50% median value and differences between these two populations are indicated by hazard ratios. P-values shown are those obtained by univariate analysis of the Breastmark database. Adjusted p-values were calculated as described in Methods. HRs that differed significantly between the high and low expressing populations are indicated by an asterisk (P≤ 0.05). ‘Events’ refers to number of patients in the sample population for whom distant metastasis occurred.

**Table 3:** Comparison of hazard ratios of the 5 genes that were most significantly associated with metastasis based on the *BreastMark* database.

Anaesthetic/Analgesic Receptor Targets	Gene	Distant Metastasis		Local recurrence	
		Hazard Ratio	Adjusted P-value	Hazard Ratio	Adjusted P-value
NMDA Receptors	GRIN3a	0.59 (0.44–0.79)	4.6x10^-3^	0.89 (0.76–1.06)	0.44
	Grina	1.353 (1.12–1.64)	0.01	1.12 (0.99–1.28)	0.22
NA Channel Transporter	SLC6A2	1.25 (1.07–1.47)	0.01	1.04 (0.93–1.17)	0.78
Glycine Receptor	GLRAB	0.69 (0.59–0.81)	1.9x10^-4^	0.87 (0.77–0.99)	0.23
GABA_A_ Receptor	GABA_A_ γ 1	0.64 (0.46–0.90)	0.05	0.98 (0.82–1.16)	0.81

Notably, when adjustment for multiple testing was performed none of the 5 were significantly associated with local recurrence.

### High gene expression and risk of DDFS (metastasis)

Risk of metastasis was linked with a high expression of some target genes and a low expression of other target genes. These links plausibly reflect the mechanism of action of anaesthetic/analgesic drugs at those receptors. Specifically, high expression of genes encoding for NMDA receptor GRINA and the Noradrenaline Transporter protein (SLC6A2) were most strongly associated with a shorter time to metastasis (Fig 1, Table 2). The NMDA receptor subunit, GRINA, contains the glutamate binding site, and has been shown to be inhibited by ketamine, nitrous oxide and xenon [14, 15]. In vitro studies have also suggested that the NMDA receptor is inhibited by intravenous local anaesthetics [16]. The Noradrenaline Transporter protein (SLC6A2) is inhibited by propofol [17]. High expression of three other genes was less strongly associated with metastasis, when we performed analysis of the *Breastmark* database, without correcting for multiple testing. These encoded the delta opioid receptor, the mu opioid receptor and the GABA_A_ receptor subunit γ3 (Fig 1, Table 2).

### Low gene expression and risk of DDFS (metastasis)

Low expression of genes encoding the NMDA receptor subunit GRIN3A, glycine receptor beta subunit and GABA_A_ subunit γ1 was strongly associated with shorter disease-free survival time or metastasis (Fig 2, Table 2). These genes encode for receptor proteins that are inhibited by clinical doses of volatile anaesthetics and activated by intravenous anaesthetics such as propofol [18]. Low expression of a gene encoding the 5HT channel transporter was also associated with metastasis when we performed univariate analysis of *Breastmark*, without correcting for multiple testing (Fig 2, Table 2).

### Analysis of prognostic marker enrichment among anaesthetic-analgesic target genes

From 10,000 randomly selected gene lists of 23 genes from the *Breastmark* database, only 700 lists contained 5 or more significant prognostic markers, equaling or exceeding the ratio we observed after correcting for multiple testing. While not reaching conventional statistical significance (p = 0.07), this suggests a trend towards anaesthetic-analgesic receptor genes being enriched for prognostic markers of metastasis. Moreover, only 494 of the 10,000 randomly selected gene lists contained 9 or more significant prognostic markers, equaling or exceeding the ratio we observed on univariate analysis of the *Breastmark* database (p = 0.049).

## Discussion

In breast cancer, metastatic disease ultimately accounts for mortality and occurs in up to 25% of patients. Here, we investigated whether genes that encode anaesthetic and analgesic target proteins are significantly associated with breast cancer metastasis. This was achieved by repurposing a vast amount of gene microarray data from published studies on breast cancer. We used the added value gene expression database *BreastMark* for this purpose, which currently combines data from 26 high-quality, published, microarray studies. *BreastMark* has been robustly validated [10] and has previously been used independently to link over-expression of the WDR5 gene to poor clinical outcome in breast cancer [19]. Importantly, it is designed for use by non-bioinformaticians and therefore makes mining of gene expression data by other scientists accessible.

Our specific interest was in the anaesthetic and analgesic drug targets because of emerging evidence that choice of anaesthetic/analgesic and anaesthetic regime during tumour resection surgery may influence the likelihood of subsequent metastasis [2–4]. Some 2,200 samples in the *BreastMark* database contain relevant data on gene expression of anaesthetic/analgesic targets in breast cancer tissue and infers protein expression levels from the associated gene expression data. It is plausible that the targets of these proteins in breast cancer tissue would be comparable to that of normal tissue although this is not confirmed. Importantly, BreastMark also supplies data on relevant clinical outcomes–namely time to metastasis and time to local recurrence. This allowed us to retrospectively determine whether the gene expression status of these drug targets in breast tumours may predict disease outcome.

Unusually for a drug class, anaesthetic drugs share only broad physiochemical characteristics, while causing similar desired therapeutic effects, such as hypnosis and amnesia, and undesirable side-effects, such as cardiovascular and respiratory depression [20]. The molecular basis of anesthetic action involves membrane proteins or ion channels [20, 21]. The resultant effect of anaesthesia is produced by either increasing the inhibition of postsynaptic excitability or by decreasing presynaptic (excitatory) neurotransmitter release. While key molecular targets involved in the mechanism of anaesthetic action have been identified, the disparity of receptor types, binding sites and low-affinity anaesthetic interactions are indicators that, the exact mechanism of action of general anaesthetics remain unclear. Furthermore, possible gene expression effects have not yet been investigated.

The primary inhibitory channels that are likely affected by anesthetics include the chloride channels relevant to the gamma-aminobutyric acid (GABA)_A_, glycine receptors and two-pore potassium (K2P) channels, including voltage-gated and adenosine triphosphate (ATP) potassium channels [20, 22, 23]. Excitatory inhibition targets N-methyl-d-aspartate (NMDA), and serotonin (5-HT3) receptors [20, 22, 23]. These target sites usually regulate the excitability of neuronal cells and can be positively or negatively modulated by anaesthetic drugs. At clinical concentrations, general anaesthetics usually inhibit excitatory receptors and enhance inhibitory receptors.

A reasonable extrapolation of prior evidence for targeted anesthetic sites involves non-specific pathways and processes downstream of cell surface receptors and ion channels, including the alteration of the chlorine’s channel conductance as well as the conductance of other channels (Ca^2+^, K^+^) through spatially dependent action in the brain, heart and the periphery. In addition, there are documented receptor-mediated channel (via cAMP) and G-protein mediated signaling cascade effects. Thus the protein-mediated effects of anaesthesia are not isolated events, but rather a concert of effects produced via alterations in ion conductance and signaling cascade effects.

The signaling pathways mediated by anaesthetic targeted receptor proteins have not been investigated as potential ‘switches’ to enhance or inhibit cancer metastasis. Given the interplay of these complex pathways, their non-specific nature, and their widespread effects on multi-organ systems, the hypothesis that these signaling pathways may influence cancer inhibition/progression would be challenging to investigate. Our current study examines a database providing gene expression information on individual genes and thus cannot infer how complexes or cascades of proteins may work. Instead this study focusses on genes encoding for specific receptor proteins that bind to anaesthetics/analgesics, occurring upstream of these signaling pathways.

To select receptor proteins for this analysis, we included those where there is evidence that either opioid analgesics, volatile or intravenous anaesthetics bind to them. Of the list of 23 genes that were compiled, 9 genes were associated with metastasis on univariate analysis and 5 remained significant upon correction for multiple testing. Within this group of five genes with strongest associations, two showed significantly reduced time to metastasis when highly expressed and 3 showed significantly reduced time to metastasis when their expression was reduced.

The noradrenaline channel transporter SLC6A2 is inhibited by propofol at clinically relevant concentrations [17]. High genetic expression of a receptor protein usually inhibited by anaesthetic propofol appears to support the previously much published and investigated hypothesis: that intravenous anaesthesia and regional analgesia may offer protective benefits compared to inhalational anaesthesia and opioid analgesia against metastasis after cancer resection surgery [2, 24].

High expression of the NMDA receptor subunit encoded by GRINA was also associated with metastasis. GRINA encodes the glutamate binding site, which has been shown to be inhibited by ketamine, nitrous oxide and xenon [14, 15]. Ketamine, is a clinically used, intravenous NMDA antagonist, which, in keeping with the hypothesis, might be expected to be associated with reduced metastasis. But existing evidence from experimental work is conflicting. Some data suggests ketamine indeed might reduce metastasis [25] others suggest it might be associated with increased metastasis [26]. Nitrous oxide (N_2_O) is also thought to act, in part, as an NMDA antagonist. While there is little experimental data on N_2_O in cancer models, a follow-up analysis of a previous randomized controlled trial showed no association between N_2_O use and cancer metastasis [27]. In vitro studies have also demonstrated that the NMDA receptor subunit GRINA is inhibited by intravenous local anaesthetics [16]. This is particularly of interest, given that strong in vitro data has already demonstrated that amide local anaesthetics can inhibit cancer cell migration [28]. Separately, amide local anaesthetics were shown to inhibit cancer cell proliferation [29].

In contrast, low expression of the NMDA receptor subunit GRIN3A was associated with metastasis. This may be attributable to the different functions of these NMDA receptor subunits. In vitro studies have shown GRIN3A to encode a cation channel with unique properties that include activation by glycine (but not NMDA), lack of permeation by Ca^2+^ and resistance to blockade by NMDA receptor antagonists [30]. The co-expression of GRIN3A appears to be required to form glycine-activated receptors in mammalian cell hosts [30, 31].

Low expression of the glycine beta receptor subunit showed the strongest association with metastasis of any of the drug targets we screened. Glycine receptors are modulated by both propofol and volatile anaesthesia [32, 33], with both types of anaesthetic exerting an effect on the alpha receptor subunits. However, the beta subunit is positively modulated by propofol [34]. This observation suggests that tumours with lower expression of this receptor may be less responsive to propofol.

GABA_A_ receptors are pentameric membrane proteins that operate GABA-gated chloride channels containing alpha, beta and gamma subunits, usually in a ratio of 2:2:1 [35]. Both the propofol and volatile anaesthetics act as positive allosteric modulators of the GABA_A_ receptor at clinically relevant concentrations, however distinct subunit binding specificities have been identified [36]. While most research has focused on the effect of different anaesthetics on the alpha and beta subunits, studies have previously demonstrated that the gamma subunits of GABA_A_ can impact and alter propofol binding [37].

Among the genes that we studied, there is most compelling evidence from previous studies to link μ opioid receptor (MOR) status to cancer outcome. However, some conflicting and inconclusive data has arisen. In non-small cell lung cancer MOR is over-expressed [38] and this is linked to tumour growth, metastasis and epithelial-mesenchymal transition [39]. In breast cancer, both growth promoting and inhibitory effects of morphine on tumour cells have been reported [40, 41] and the common A118G genetic polymorphism in the MOR that is linked to reduced analgesic response has, contradictorily, been correlated with both decreased breast cancer-specific mortality [42] and increased breast cancer incidence [43]. However, to date no clinical studies have linked metastasis in breast cancer to opioid gene expression levels. Also, to our knowledge there is no evidence that expression of other opioid receptors (eg: delta and kappa) are linked to cancer metatastasis. Analysis of *Breastmark* indicated that high of expression of both the MOR and delta opioid receptors was associated with metastasis, whereas no such association was observed for the kappa opioid receptor.

When considered as a group of genes, our analysis identified a trend (p = 0.07) that suggests that the proteins encoded by anaesthetic-analgesic target genes might be enriched for prognostic indicators of metastasis. While 9 of 23 genes were significantly associated with metastasis on analysis of *BreastMark* and 5 remained associated with metastasis when a stringent correction for multiple testing was applied.

By using a meta-analysis approach, the *Breastmark* database is large and well-validated. However, there are limitations to this study. It is based on meta-analysis of retrospective data from surgically excised breast cancer tissue, and we cannot account for potential clinical confounding factors that may be present. While it is intuitive to suggest that tumours may respond differently to anaesthetic or analgesic agents given during surgery depending on their pharmacogenetic expression profiles, there is no information on the anaesthetic or analgesic technique used during surgical excision of the breast cancer tissue analysed within the *Breastmark* database.

It would be beneficial to compare the ratio of these genes expressed in breast cancer tissue versus matched normal breast tissue, however the datasets incorporated in BreastMark (26 in total), do not contain matched normal breast tissue as it was not common practice to collect and microarray normal adjacent tissue when these datasets were collated.

It is plausible that any potential effect of anaesthetic drugs on cancer recurrence may be mediated by receptors other than those which mediate the hypnotic, amnesic and analgesic effect of these drugs, yet the association between MOR and greater metastatic recurrence in some tumours (notably lung) suggests that the action of anaesthetic-analgesic drugs through their predicted target receptors might be influential in cancer recurrence [9]. MOR expression occurs in breast tumours and preliminary data suggests it may be mediated by anaesthetic technique (Levins K et al, manuscript in peer review).

## Conclusions

In summary, in this analysis of the expression of anaesthetic-analgesic targets in excised breast cancer tissue, several anaesthetic-analgesic receptor genes were associated with metastatic spread in breast cancer. Overall there was no significant enrichment in prognostic markers of metastasis, although a trend was observed.
